# An interpretable semi-supervised framework for patch-based classification of breast cancer

**DOI:** 10.1038/s41598-022-20268-7

**Published:** 2022-10-06

**Authors:** Radwa El Shawi, Khatia Kilanava, Sherif Sakr

**Affiliations:** Institute of Computer Science, Tartu University, Tartu, Estonia

**Keywords:** Computational models, Image processing, Machine learning, Statistical methods, Health care

## Abstract

Developing effective invasive Ductal Carcinoma (IDC) detection methods remains a challenging problem for breast cancer diagnosis. Recently, there has been notable success in utilizing deep neural networks in various application domains; however, it is well-known that deep neural networks require a large amount of labelled training data to achieve high accuracy. Such amounts of manually labelled data are time-consuming and expensive, especially when domain expertise is required. To this end, we present a novel semi-supervised learning framework for IDC detection using small amounts of labelled training examples to take advantage of cheap available unlabeled data. To gain trust in the prediction of the framework, we explain the prediction globally. Our proposed framework consists of five main stages: data augmentation, feature selection, dividing co-training data labelling, deep neural network modelling, and the interpretability of neural network prediction. The data cohort used in this study contains digitized BCa histopathology slides from 162 women with IDC at the Hospital of the University of Pennsylvania and the Cancer Institute of New Jersey. To evaluate the effectiveness of the deep neural network model used by the proposed approach, we compare it to different state-of-the-art network architectures; AlexNet and a shallow VGG network trained only on the labelled data. The results show that the deep neural network used in our proposed approach outperforms the state-of-the-art techniques achieving balanced accuracy of 0.73 and F-measure of 0.843. In addition, we compare the performance of the proposed semi-supervised approach to state-of-the-art semi-supervised DCGAN technique and self-learning technique. The experimental evaluation shows that our framework outperforms both semi-supervised techniques and detects IDC with an accuracy of 85.75%, a balanced accuracy of 0.865, and an F-measure of 0.773 using only 10% labelled instances from the training dataset while the rest of the training dataset is treated as unlabeled.

## Introduction

According to the American National Breast Cancer Organization, breast cancer is the second leading cancer type that causes death among women^1^. Breast cancer contributes to around 25% of all types of cancers diagnosed in women. Furthermore, it contributes to 15% of cancer deaths in women^2^. Invasive Ductal Carcinoma (IDC) is the most common type of breast cancer. Among all the patients of breast cancers, around 80% are diagnosed as invasive ductal carcinomas^3^. Breast masses are the most significant findings among different types of breast abnormality. In addition, morphological features of tumour shape play vital roles in the diagnosis of tumour malignancy^4^.

Because of its high performance, deep learning has been used extensively in various application domains, including medical diagnosis, image recognition and image classification^5–11^. Nowadays, developing high quality deep learning models has become a commodity thanks to the availability of several open-source machine learning frameworks such as TensorFlow^12^ and PyTorch^13^. One of the main challenges for deep learning models is that they require vast amounts of labelled data to fine-tune their architecture and parameters. In practice, these labelled data is expensive and hard to obtain, especially in critical domains such as the medical domain. In addition, developing a well-performing deep neural network architecture and fine-tuning its hyper-parameters is a very challenging and time-consuming task due to the vast search space. In particular, the performance of deep neural network architecture can significantly vary with different hyper-parameter values. Therefore, Neural Architecture Search (NAS) has become an essential technique for automating the process of finding the best performing neural network architecture along with the best set of their hyper-parameter values. In practice, NAS has been successfully used to design the model architecture for various image classification and language processing tasks^14–17^.

Generative Adversarial Networks (GANs)^18^ is a special type of neural network that consists of two main components: *generator* and *discriminator*. Both generator and discriminator are neural networks in which the generator focuses on generating images while the discriminator focuses on discriminating between the synthetic generated images and the original ones. Recently, GANs have received huge attention due to their capability of data generation without explicitly modelling the probability density function. They have shown to be successful in different domains and have achieved state-of-the-art performance in many image generation tasks, including classification, super-resolution^19^ and image-to-image translation^20^. Therefore, GANs have been widely adopted in the medical domain^21^ to tackle the privacy concerns related to medical image diagnosis, in addition to the limited number of positive cases of each pathology. Furthermore, the lack of sufficiently labelled medical images poses another challenge for adopting the traditional supervised training techniques and motivates approaches that incorporate unlabeled data that might be available. These approaches include *semi-supervised learning* and *transfer learning*^22^. There has been a lot of research that examines the use of generative models in the semi-supervised setting. Salimans, et al.^23^ presented a technique to utilize GANs for solving classification problems with *k* classes. More specifically, they extended vanilla GAN such that the set of labelled examples is augmented with the generated samples from the generator (fully connected network). The discriminator is modified in a way to predict $k+1$ classes (the original *k* classes plus the fake generated class from the generator). Adiwardana et al.^24^ utilizes the GANs as in ^23^ but replaced the fully connected generator network with Deep Convolutional Generative Adversarial Network (DCGAN)^25^. Such change resulted in a significant performance boost in supervised image recognition tasks using a small amount of labelled data. On another hand, *transfer learning* tries to gain performance from a larger labelled dataset for a related task. It has been empirically observed that features learned from enough training examples by deep learning models can generalize to other related problems. In the computer vision domain, it is a common practice to reuse layers from large pre-trained networks such as VGG^26^ and Inception^27^.

A successful methodology for semi-supervised learning is based on obtaining one or more enlarged labelled dataset(s) to classify unlabelled data based on the most confident predictions^28^. Self-labeled techniques are typically divided into *self-training* and *co-training*. *Self-training* techniques are the most basic of pseudo-labelling approaches^29^. They consist of a single supervised classifier that is iteratively trained on both labelled data and data that has been labelled in previous iterations of the algorithm. At the beginning of the self-training process, a classifier is trained on the labelled data, and then this classifier is used to obtain predictions for the unlabelled data. Then the most confident predictions are then added to the set of labelled data and the supervised classifier is retrained on both the originally labelled and the newly obtained pseudo-labelled data. The process is typically repeated until no more unlabelled data remain. *Co-training*^30^ is one of the most popular techniques for semi-supervised learning in which two classifiers are trained by labelling the unlabeled data for the other classifier and then making the final decision for a particular instance based on the agreement of the two classifiers. It assumes that each sample is described using two feature views, each of which provides separate, complementary information about the sample. Ideally, the two views are independent, and each view is sufficient, such that the label of a sample can be predicted independently from each view. Co-training first learns a separate model for each view using a small amount of labelled data. Then, the samples with the most confident predictions of each model on the unlabeled data are added to the labelled data iteratively. Co-training has been proven to be beneficial in a variety of application domains email classification^31^, sentiment classification^32^, web page mining^30^ and visual tracking^33,34^. For example, Wan^35^ proposed a co-training strategy to solve the cross-lingual sentiment classification problem by treating English and Chinese features as two independent views. Li et al.^32^ studied the problem of semi-supervised learning for imbalanced sentiment classification using a dynamic co-training method. This method relies on different views generated from various random feature subspaces, which were dynamically generated to deal with the imbalanced class distribution problem. Notably, there is a limited application for co-training in the area of image classification mainly because obtaining two independent, and sufficient representations of a single image is quite challenging. Nevertheless, some recent studies have relaxed the independence assumption^36–39^. These studies showed that it is enough for the success of the co-training algorithm to have at least some cases when the classifier on one representation makes confident decisions while the classifier on the other representation does not have much confidence in its own decision. This weaker expanding property of the co-training algorithm has been well demonstrated in different studies^36,39,40^. These studies showed that a random split of a nature single feature set usually contribute to the success of the co-training algorithm in^38^ where the authors proposed an elegant algorithm to automatically decompose a single feature set into two complementary subsets as inputs of the co-training algorithm.

Although deep neural networks have been well-performing in various application domains^41^, however, in the medical domain, physicians still find it hard to trust the prediction of these black-box models and hence prefer white-box models even if they achieve lower performance compared to black-box models^42^. Since May 2018, machine learning interpretability has received lots of attention, especially due to the General Data Protection Regulations (GDPR) ’*right to explanation*’^43^. In particular, the GDPR requires all decisions made automatically to be explained as a safeguard for the rights and freedom of EU citizens^44^. One way to define machine learning interpretability is the ability to understand and comprehend the decision made by a machine learning model^45^. In general, machine learning interpretability techniques can be broadly categorized into *global* and *local* techniques^46–50^. Global interpretability techniques focus on explaining the model globally and enable users to comprehend an aspect of the whole model at once^47^. On the other hand, local techniques focus on explaining the prediction of a single instance. One common way for local interpretability is saliency methods that have been used intensively in several gradient-based methods^51–54^. The output of saliency methods shows the importance of individual outcomes as an overlay on the input image to be explained. Such approaches suffer from being limited and inconsistent to some extent^55,56^. Another line of research shows that linear classifiers can learn meaningful directions that can be mapped to semantically meaningful word embedding^57^ or visual concepts^58,59^.

### Motivation and contribution

In this paper, we hypothesize that combining a small amount of labelled data with a large amount of unlabelled data is one effective way of combatting the scarcity of labelled data and, hence, effectively enabling the use of deep learning models. Motivated by the current trend for favouring complex machine learning models at the expense of interpretability, we explain the predictions of our proposed framework globally to provide physicians with complementary insights about the model. In particular, the main contribution of this paper is summarized as follows:

- We developed a semi-supervised deep learning framework for IDC detection using a small number of labelled data combined with a large number of unlabelled data. The proposed framework outperforms the state-of-the-art semi-supervised DCGAN technique and self-learning technique achieving a balanced accuracy of 0.865, an accuracy of 85.75%, and an F-measure of 0.773.
- We used AutoML technique to design a deep neural network architecture that outperforms the state-of-the-art performance for IDC detection on digitized BCa histopathology slides obtained from 162 women who have been diagnosed with IDC at the Cancer Institute of New Jersey and the hospital of the University of Pennsylvania.
- We interpret the predictions of our semi-supervised model globally by learning meaningful high-level concepts and using directional derivatives to quantify the degree to which such concepts are essential to the IDC prediction.

Ensuring repeatability is one of the main targets of this work. Therefore, we provide access to the source codes and the detailed results for the experiments in our project repository^60^. The remainder of this paper is organized as follows. In "Methods" section describes the building blocks for our interpretable semi-supervised deep learning framework for IDC detection. The details of our experimental evaluation are described in "Experimental evaluation" section. We discuss our results in "Discussion" section before we conclude the paper in "Conclusion" section.

## Methods

**Figure 1:**
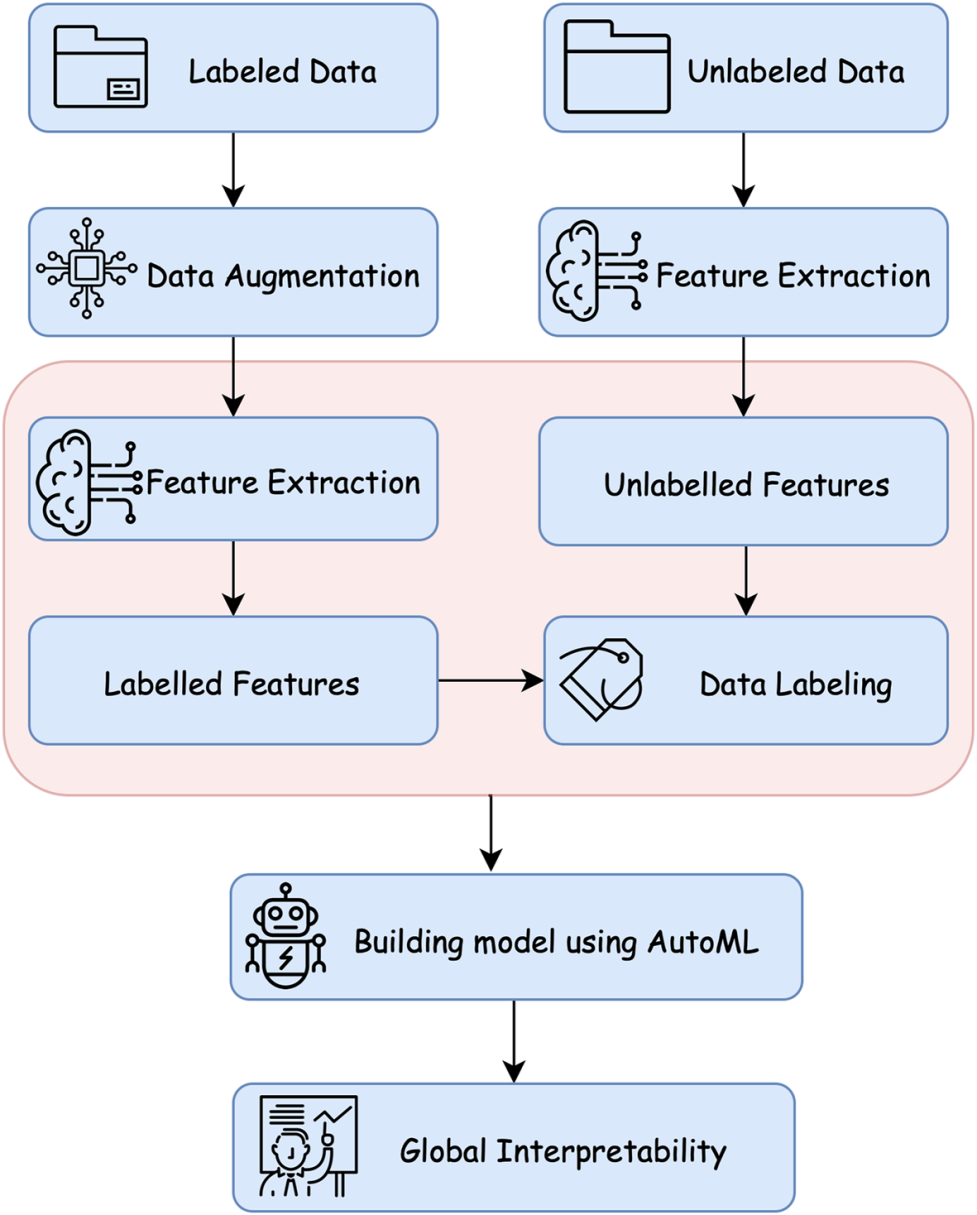
Flowchart of the proposed framework.

Figure 1 illustrates the architecture of our framework that consists of five main stages including *data augmentation*, *feature selection*, *dividing co-training data labeling*, *deep neural network* and *interpretability* of the neural network predictions. In the following subsections, we explain the different building blocks of our architecture.

### Datasets

For IDC detection, we use a data cohort that consists of digitized Bca histopathology slides obtained from 162 women diagnosed with IDC at the Cancer Institute of New Jersey and the hospital of the University of Pennsylvania. The cohort was randomly split into three subsets, including 84 for training, 29 for validation and 49 for evaluation. The patch-based dataset used in this study obtained from the original cohort consists of 82,883 patches for training, 31,352 patches for validation (i.e. 114,235 patches for full training) and 50,963 instances for testing^61^. From the 114,235 patches of the entire training dataset, we randomly select 12k instances (6k instances from each class) as training data denoted $D_{train}$, and we remove the ground truth of the rest of the entire training patches obtaining 102,235 unlabelled patches. For more information about the dataset, we refer the interested readers to^62^.

The concepts used by the global interpretability technique are extracted from the nuclear segmentation dataset^63^. The nuclear segmentation dataset consists of 30 whole slide images of digitized tissue samples of several organs obtained from 18 different hospitals. The dataset contains nuclear appearances of seven different organs, including breast, liver, kidney, prostate, colon, stomach, and bladder. Since computational requirements for processing WSIs are high, a sub-image of 1000$\times$1000 is cropped from WSIs, and more than 21,000 nuclear boundaries are annotated in Aperio ImageScope^64^. Only five images are extracted that contain the nuclear appearance of breast cancer which are then segmented into 100 patches in which the concepts are extracted.

### Data augmentation

Data Augmentation is a widely used technique in various deep learning approaches in the presence of a limited amount of labelled training instances to reduce overfitting and improve the accuracy and the robustness of a classifier. In this work, we employ DCGAN to generate synthetic patches. The DCGAN generator consists of a fully connected layer projecting an input of 100-dimensional uniform distribution to four convolution layers with filter sizes of 256, 128, 64 and 32 and kernel size of 5$\times$5. Except for the output layer, the used activation function is rectified linear unit (Relu). Batch normalization is performed on all layers except for the last one. We train a DCGAN on $D_{train}$ as a preprocessing step. The DCGAN is trained separately on each label using multi-channel image patches containing both the acquired image and the ground truth label. The number of synthetic examples generated for each class is 6k patches. Such 12k generated synthetic patches, denoted $D_{GAN}$, are then used to augment $D_{train}$. DCGAN is trained using Stochastic Gradient Descent as an optimizer for 1200 epochs with the *batch size* of 16 and *learning rate* equal to 0.0003. The loss function applied is a binary cross-entropy. Parameters are the same for both discriminator and generator.

### Feature selection

Complex deep convolution neural network architectures that contain millions of parameters such as Inception, ResNet and VGG have achieved state-of-the-art performance in different applications^27^. Training these networks requires a large amount of data, as training with only a small amount of data may lead to overfitting. When the size of the training dataset is too small, it makes the noises have a great chance of being learned and later act as a basis for predictions^65^. One approach that is commonly used with such networks with a limited number of training examples is *fine-tuning*, in which only part of the pre-trained neural network is being fitted on the new dataset. In our experiments, we have considered this approach. Inspired by^66^, in this work, we use a standard pre-trained VGG-16 network for feature extraction. We follow the same procedure for extracting features from both labelled and unlabelled data. We remove the fully connected layers from the VGG network and apply the Global Average Pooling operation to the four internal convolutions layers with 128, 256, 512, and 512 channels, respectively. Next, we concatenate them to form one vector of length 1408.

### Data labeling

The original co-training process introduced by Blum and Mitchell^30^ starts with two independent attribute subsets, and the unlabelled data is labelled once the two classifiers reach an agreement. It has been shown that the independence assumption can be relaxed, and the co-training is still powerful under a weaker independence assumption^67^. Since the semi-supervised training is very sensitive to the initial labelled dataset, we develop a co-training procedure to achieve confident labelling. Since the size of the labelled dataset is relatively small, partitioning it into disjoint subsets will result in too small subsets to build a reliable model^68^. First, our procedure starts by shuffling $D_{GAN}$ and $D_{train}$ into two equal-sized subgroups five times so that the size of each subgroup is half the size of $D_{train}$. Next, the 10 subgroups of $D_{train}$ and $D_{GAN}$ are used to train 10 gradient-boost classifiers to label the unlabelled data and the common confident data, denoted $D_{conf}$, are then appended to $D_{train}$ and $D_{GAN}$. Instances in $D_{conf}$ are chosen by setting a threshold of the minimum number of classifiers that should agree on the predicted label of the unlabelled instance. Such threshold is set to 5. The process is repeated until no more common confident instances appeared. Note that our approach only adds $D_{conf}$ to the $D_{train}$ and $D_{GAN}$; not all unlabelled data are added eventually to the labelled dataset, and hence the instances that are not added are considered noisy data and are not considered in building the final model. Note that $D_{GAN}$ is only used in the labelling process and is not considered in training the final network. So, the final dataset from this stage which is used to train our final model consists of $D_{train}$ and $D_{conf}$.

### Deep convolution neural network model

**Figure 2:**
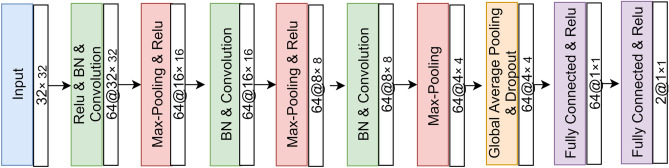
The structure of the CNN used in this study.

In general, different neural network architectures can have significantly different performance results. In practice, most of the currently employed network architectures are manually developed by human experts. Developing such network architectures is a very time-consuming and error-prone process^69^. In this work, we use Neural Network Intelligence(NNI)^70^, an open-source toolkit by Microsoft for automated machine learning, to find the best network architecture along with the best set of hyper-parameters. NNI accelerates and simplifies the whole search space using a built-in super-parameter selection algorithm. For hyper-parameter optimization, we chose to use the Tree-structured Parzen Estimator^71^ (TPE). Table 1 shows the hyper-parameter configuration space of NNI. The architecture of the neural network found by the NNI is illustrated in Fig. 2. The network consists of three convolution layers of sizes $32\times 32$, $16\times 16$, and $8\times 8$, respectively. Batch-normalization (BN) is applied before each convolution layer. For each convolution layer, 64 kernels were applied for the previous feature maps. Each of the convolution layers is followed by a max-pooling layer. The first two max-pooling layers are passed through the Relu non-linear activation function. The last pooling layer is of size $4\times 4$ and is followed by the global average pooling layer, followed by a dropout layer. Following the global average pooling layer is two fully connected layers passed by Relu non-linear activation function. The last fully-connected layer generates the final membership degree for each class. The parameters used in the network are as follows: *learning rate* = 0.01, *number of epoch* = 80, *batch size* = 32, and *decay* = $1e^{-5}$. For finding the best network architecture and hyper-parameters, the NNI has been assigned a budget of 48 hours on GPU environment.

**Table 1:** Search space of NNI.

Hyper-parameters	Search space
Optimizer	{Adam, SGD, Adamax, RMSprop}
Learning rate	{0.001, 0.002,..., 0.1}
Decay	{0.00001, 0.00002,..., 0.9}
Batch size	{32, 64, 128, 256, 512}
Activation function	{Relu, Softplus, Tanh, LeakyReLU}
Dimensionality of the last hidden layer	{8, 16, 32, 64, 128, 256, 512}
Kernel size of convolutional layers	{4, 8,.., 64}
Number of kernels	{1, 2, ..., 100}

### Time complexity analysis

In this section, we analyze the time complexity of the proposed model. Let us first consider the time complexity of the data augmentation stage. Training DCGAN on $D_{train}$ takes $O(|D_{train}|Td)$, where *T* is the number of iterations and *d* is the patch size^72^. The complexity of the feature extraction phase using VGG16 for both labelled and unlabelled data is as follows. Convolutional and fully connected layers are the most time-consuming parts of VGG16. Thus, we will focus on the time complexity of these two kinds of layers. Since we removed all fully connected layers, then we only include the complexity of the convolution layers. Let $f_l$ be the number of input channels of the $l-th$ convolutional layer, $n_l$ be the number of filters/channels in the l-th convolutional layer, $s_l$ be the spatial size of the filter and $m_l$ be the spatial size of the output feature map. Then, updating filter weights of $l-th$ convolution layer for one input costs is $O(f_ls^{2}_l n_lm^{2}_l)$^73^. Note that the filter size of VGG16 is fixed ($s_l=3$). Since we fix the first *L* convolutional layers and only fine-tune weights of the last $13-L$ layers, then the total cost is $O(I.P \sum ^{13}_{l=L+1} (f_{l}n_{l}m^{2}_{l}))$, where *I* is the number of iterations and *P* is the size of the whole training dataset including the labelled and unlabelled data (114,235 patches). For the data labelling phase, training 10 gradient-boost classifiers on $D_{train}$ and $D_{GAN}$ takes $O(th||x||_0log|D_{train}+D_{GAN}|)$, where *t* is the number of trees, *h* is the maximum depth of the trees, and $||x||_0$ is the number of non-missing entries in the training data^74^. Using the 10 gradient-boost classifiers to label the unlabelled data takes $O(|D_{unlabelled}|th)$, where $D_{unlabelled}$ is the set of the unlabelled instances. The time complexity for training the deep convolution neural network in "Deep convolution neural network model" section on $D_{train}$ and $D_{conf}$ is dominated by the time complexity of the convolutional and fully connected layers. The time complexity of the convolutional layers for each iteration is $O(|D_{train}+D_{conf}|\sum ^{3}_{l=1} f'_{l}n'_{l}m'^{2}_{l}s'^{2})$, where $f'_l$ be the number of input channels of the $l-th$ convolutional layer, $n'_l$ be the number of filters/channels in the l-th convolutional layer, $s'_l$ be the spatial size of the filter and $m'_l$ be the spatial size of the output feature map. The time complexity of the fully connected layers for each iteration is $O(|D_{train}+D_{conf}|m'^2_{2}n'_{3}d')$+$|D_{train}+D_{conf}|O(d'^2)$, where $d'$ is the dimension of the vector. Hence, the time complexity for training the deep convolution neural network is $O(I'|D_{train}+D_{conf}|[\sum ^{3}_{l=1} (f'_{l}n'_{l}m'^{2}_{l}s'^{2})+m'^2_{2}n'_{3}d'+d'^2])$, where $I'$ is the number of iterations. We can conclude that training time complexity of data augmentation, feature extraction, data labeling, and deep neural network stages can roughly be given as $O(|p|.[Td+I. \sum ^{13}_{l=L+1} (f_{l}n_{l}m^{2}_{l})+th||x||_{0}log|p|+I'.\sum ^{3}_{l=1} (f'_{l}n'_{l}m'^{2}_{l}s'^{2}) +m'^2_{2}n'_{3}d'+d'^2])$; which is linear in the size of the whole training dataset.

### Global interpretability

In this work, we use a global interpretability technique which is based on Concept Activation Vector (CAV) and can provide an interpretation of the internal state of neural networks using real human-friendly concepts^75,76^. Such concepts are tied to real-world data that represents interesting and relevant concepts. Testing the concept activation vector uses directional derivatives to quantify the degree of importance of a particular concept to the model prediction. Informally, the key idea of the technique relies on the evidence that a feedforward neural network works by gradually disentangling a particular concept across layers^77^. Such learnt concept is not necessary acquired by a single neuron but more generally in linear combinations of neurons^78,79^. Hence, the space of neurons activations in neural network layers can have a meaningful global linear structure. Such structure can be uncovered by training a model that can map the representation in a single network layer to meaningful user-defined concepts. We adopted the technique of Graziani et al.^76^ which is summarized as follows. In this approach, the first step is to define the interesting concepts relevant to IDC prediction. Nuclear morphometric and appearance features such as average size and pleomorphism can help in assessing cancer grades and predicting treatment^80,81^. In this study, we use concepts by referring to the Nottingham Histologic Grading system (NHG)^82^. Such concepts are extracted from the nuclear segmentation dataset^63^ that quantify the impact of variations in nuclei size, area and texture. The second step is to compute the Pearson product-moment correlation coefficient $\rho$ between each concept and the network prediction for each input patch. If the correlation coefficient for a particular concept is low, this concept is not relevant to the model prediction. A high correlation value for a particular concept refers to whether such concept is positively or negatively affecting the prediction. We repeat the following steps for each concept of interest. Let $\phi _{l}(x)$ be the activations for input patch *x* at layer *l*. The third step is to find a unit vector $v_{c}^{l}$ in the space of activations of layer *l* in the network that represents the increasing direction for a particular concept of interest. Such vector is computed as the least-squares linear regression fit $\{{\phi _{l}(x_i),c_i}\}$ on the nuclear segmentation dataset, where $c_i$ is the concept measure for a particular concept *C* for input image $x_i$. The fourth step is to calculate the sensitivity to changes in each input $x_i$ along the direction of the increasing values of the concept measures at neural network activation layer *l*. The sensitivity score $S_{C,l,i}$ is calculated as the directional derivative along the direction of $v_{c}^{l}$.

1 $$\begin{aligned} S_{C,l,i}=\frac{\partial f(x_i)}{\phi _{l}(x_i)}\cdot v_{c}^{l} \end{aligned}$$

where $f(x_i)$ is the network prediction for instance $x_i$. The sensitivity sign is interpreted as the direction of the change, whereas the magnitude of the sensitivity reflects the rate of change. In this work, we use the bidirectional relevance score *Br*^76^ that is defined as the ratio between the coefficient of determination of the least-squares regression, $R^2$, and the coefficient of variation $\frac{\sigma }{\mu }$ of the sensitivity scores calculated in the previous steps over all the test instances in the testing dataset.

2 $$\begin{aligned} Br=R^2\times \frac{\sigma }{\mu } \end{aligned}$$

Where $\sigma$ and $\mu$ are the standard deviation and the mean of the sensitivity scores, respectively. The *Br* scores are calculated for all of the concepts of interest and scaled over the range of $[-1,1]$.

## Experimental evaluation

### Experimental setup

We conducted our experiments on two hardware environments: a CPU environment and a GPU environment. The CPU environment runs on CentOS release 7.5.1804 with 64 core Intel Xeon Processor (Skylake, IBRS) @ 2.00GHz;240 GB DIMM memory; and 240 GB SSD data storage. The GPU experiments are performed on a single machine running on Debian GNU/Linux 9 (stretch) with an 8 core Intel(R) Xeon(R) CPU @ 2.00GHz; NVIDIA Tesla P4;36 GB DIMM memory; and 300 GB SSD data storage.

### Results

#### Data augmentation

**Figure 3:**
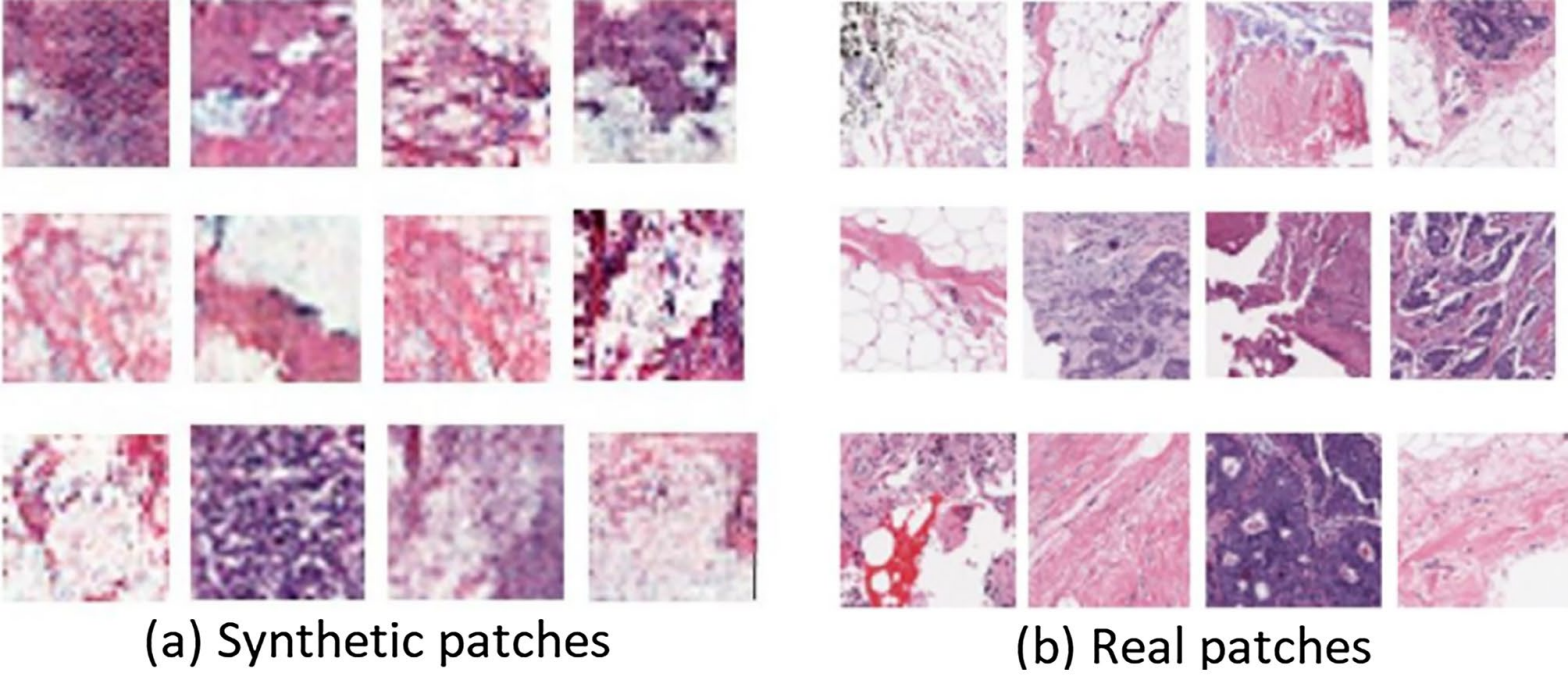
Examples of real and GAN generated synthetic patches.

**Figure 4:**
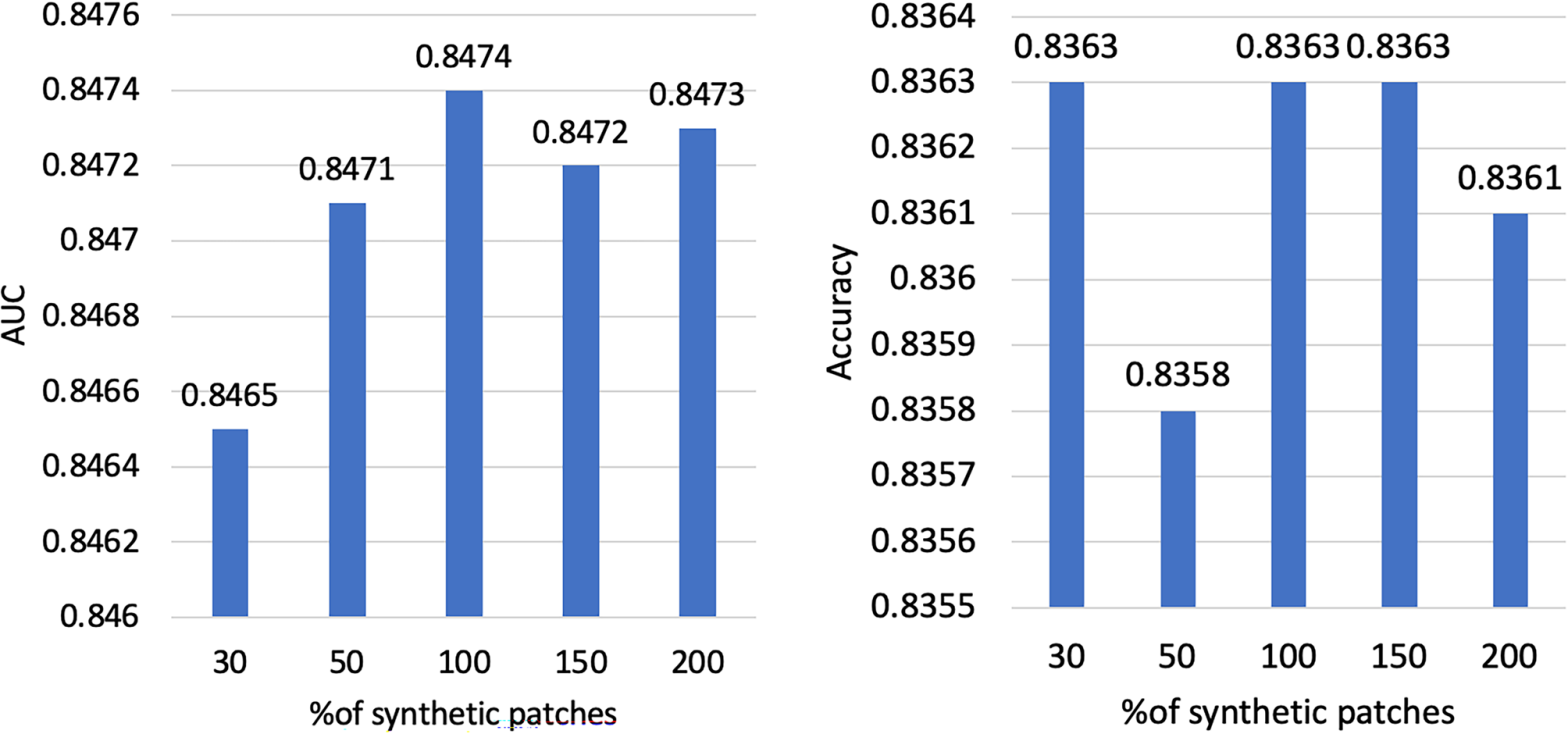
The data labeling performance with varying amounts of additional synthetic data.

Examples of real and synthetic patches generated by DCGAN are illustrated in Figure 3. The data labelling performance of using only $D_{train}$ and varying amounts of additional synthetic data is shown in Figure 4. The performance is measured by calculating the accuracy and the AUC of the newly labelled data. The labelling model remains unchanged when examining the effect of different amounts of synthetic patches generated by the DCGAN. This provides a fair platform to observe the impact of GAN augmentation by ensuring that any changes in performance are due to the additional synthetic patches and not due to changes in the labelling model itself. Notably, increasing the amount of synthetic generated patches to 100% increases the labelling performance to an accuracy of 83.63% and an AUC of 0.8474 compared to an accuracy of 81.51% and an AUC of 0.815 without using synthetic patches. The labelling performance slightly drops when increasing the percentage of synthetic generated patches to 150% and 200% as shown in Figure 4.

**Figure 5:**
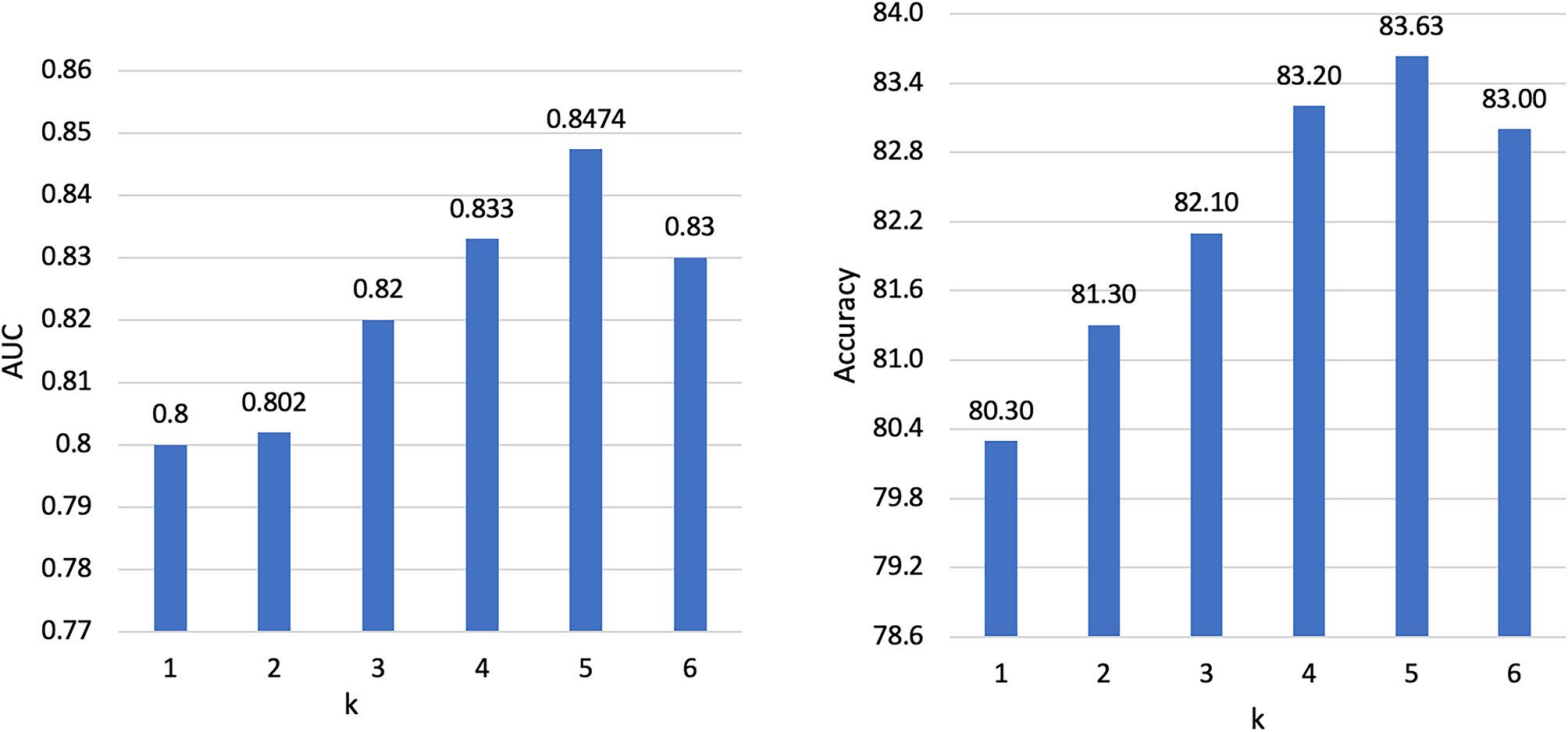
The data labelling performance with varying number of bootstrapping samples (k).

#### Data labeling

For ablation analysis for the co-training technique used in the data labelling phase in our proposed semi-supervised approach, we evaluate the effect of using different partitioning strategies for $D_{train}$ and $D_{CGAN}$ on the labelling performance. More specifically, we examine the labelling performance when partitioning $D_{train}$ and $D_{CGAN}$ into two equal-sized *k* subgroups where $k = {1, 2,.., 6}$, such that the size of each subgroup is half the size of $D_{train}$. Next, we report the performance of 2*k* gradient-boost classifiers trained on 2*k* subgroups of labelled data to label the unlabelled data (See Fig. 5). We set the threshold of the minimum number of classifiers to agree on the predicted label of the unlabelled instance to be *k*. Notably, increasing the amount of bootstrapping samples to 10 ($k=5$) achieves the highest performance, while the performance drops slightly when increasing the number of bootstrapping samples to 12 ($k=6$), as shown in Fig. 5.

Table 2 reports ablation experiments to evaluate the impact of using different classifiers in the co-training technique used in the data labelling phase of our proposed semi-supervised approach. More specifically, we partitioned $D_{train}$ and $D_{CGAN}$ into two equal-sized five subgroups, such that the size of each subgroup is half the size of $D_{train}$. Next, the 10 subgroups of labelled data are used to train 10 gradient-boost (GB), 10 random forest (RF), 10 decision tree (DT), 10 naive Bayes and 10 support vector machine (SVM) classifiers. For each column metric in Table 2, we highlighted the highest performance in bold font and underlined the lowest performance. The results show that gradient-boost achieves the highest performance (accuracy = 83.63%, AUC = 0.8474) while support vector machine achieves the lowest performance (accuracy = 78.35%, AUC = 0.758). The Wilcoxon signed-rank test^83^ was conducted to determine if a statistically significant difference in terms of the performance exists between gradient boost classifiers and the other classifiers. The results showed that the difference in performance is statistically significant with more than 95% level of confidence (*p*-value = 0.002).

**Table 2:** Ablation study on the impact of using different numbers of different classifiers in the proposed co-training process on the labeling performance.

Classifier	Accuracy	AUC
RF	81.21 ${\pm }$ 0.001	0.8069 ${\pm }$ 0.002
GB	**83.63** ${\pm }$ **0.001**	**0.8474** ${\pm }$ **0.001**
DT	80.01 ${\pm }$ 0.001	0.7981 ${\pm }$ 0.003
SVM	78.35 ${\pm }$ 0.012	0.758 ${\pm }$ 0.011
NB	79.01 ${\pm }$ 0.012	0.7704 ${\pm }$ 0.013

Bold entry highlights the best-performing technique.

Underlined entry highlights the worst performing technique.

#### Feature extraction

**Figure 6:**
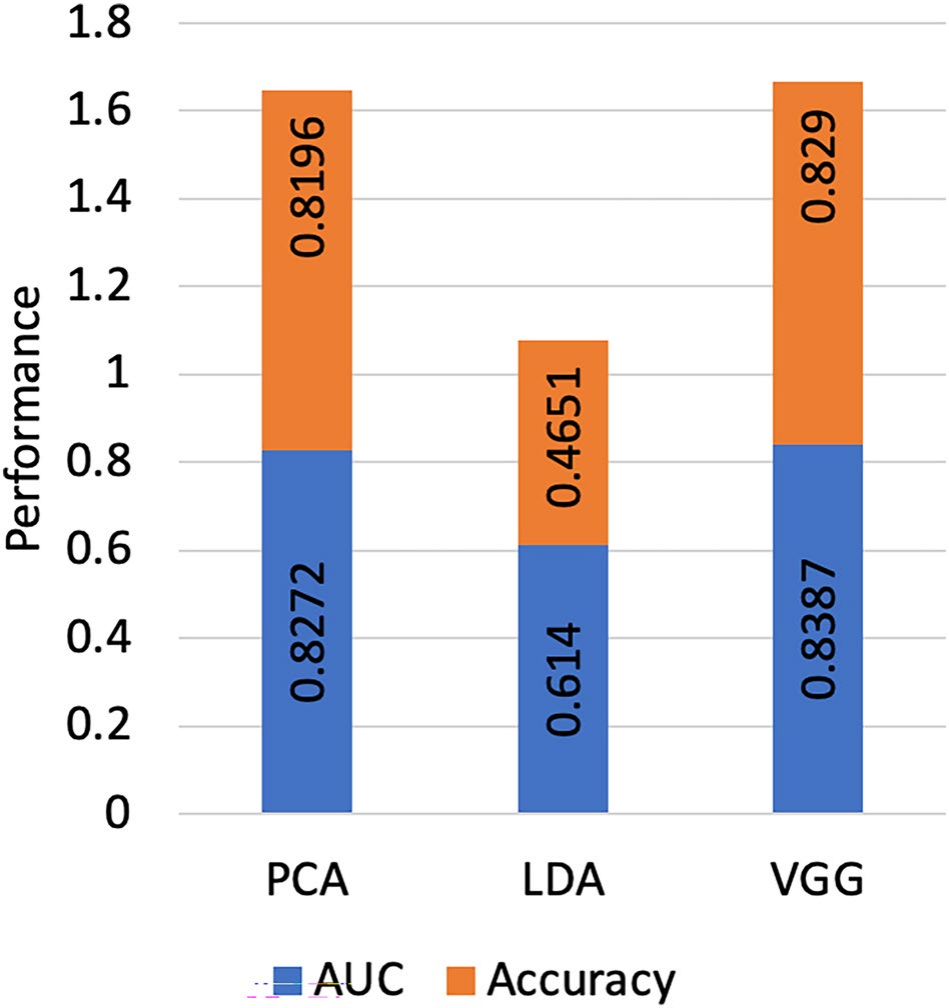
Comparison of the data labeling performance of different feature extraction techniques.

We first evaluate the impact of three different feature extraction techniques, including principal component analysis (PCA), Linear discriminant analysis (LDA) and VGG. The performance of these techniques is measured by calculating the labelling performance of the newly labelled data without including $D_{GAN}$. Table 3 shows the data labelling performance (accuracy and AUC) of PCA and LDA tested at reduced dimensions of 50, 100, 200 and 250. As shown in Table  3, LDA achieves the same performance across all tested dimensions, and that is mainly because the number of feature projections outputs by LDA is at most equal to the number of classes - 1. PCA has been shown to achieve the best performance at 100 components with an AUC of 0.8072 and accuracy of 80.96%. It worth mentioning that on average the time taken for feature extraction using PCA is almost 0.85 the time taken using LDA approach. Figure 6 shows the labelling performance of the newly labelled data using PCA, LDA, and VGG-16. The labelling performance using VGG-16 features outperforms other techniques achieving an AUC of 0.815 and accuracy of 81.51%.

**Table 3:** The performance of newly labelled data using LDA and PCA feature extraction techniques.

		Dimension			
		50	100	200	250
PCA	AUC	0.8069 ${\pm }$ 0.001	0.8072 ${\pm }$ 0.013	0.8047 ${\pm }$ 0.011	0.8041 ${\pm }$ 0.012
	Accuracy	80.87 ${\pm }$ 0.001	80.96 ${\pm }$ 0.010	79.78 ${\pm }$ 0.012	80.58 ${\pm }$ 0.014
LDA	AUC	0.6412 ${\pm }$ 0.014	0.6412 ${\pm }$ 0.011	0.6412 ${\pm }$ 0.012	0.6412 ${\pm }$ 0.013
	Accuracy	46.51 ${\pm }$ 0.012	46.51 ${\pm }$ 0.013	46.51 ${\pm }$ 0.013	46.51 ${\pm }$ 0.014

#### Deep convolution neural network model

To examine the performance of the neural network architecture and the hyper-parameters found by NNI, we run three different experiments. The first one is just feeding the whole training and validation datasets (114,235 labelled patches) and testing the network’s performance on the testing dataset (50,964 patches) averaged over 100 runs. Quantitatively, we present the F-score and the balanced accuracy for our method compared to Curz et al.^62^, and Janowczuk et al.^84^ in Table 4. To provide a fair comparison, we use the same training, validation and testing splits used by the baselines^62,84^. For each metric (column), we highlighted the highest value in bold font. The performance of the network found by NNI achieves a balanced accuracy of 0.8696 and F-measure of 0.7923 and outperforms the customized deep neural network obtained by Cruz-Roa et al.^62^, and the customized AlexNet architecture^10^ used by Janowczyk et al. ^84^.

**Table 4:** Performance comparison between our network and other approaches.

	F-measure	Balanced accuracy
Curz et al.^62^	0.7180 ${\pm }$ 0.016	0.8423 ${\pm }$ 0.018
Janowczuk et al.^84^	0.7648 ${\pm }$ 0.003	0.8468 ${\pm }$ 0.004
Our approach	**0.7923** ${\pm }$ **0.001**	**0.8696** ${\pm }$ **0.002**

Significant values are in bold.

**Figure 7:**
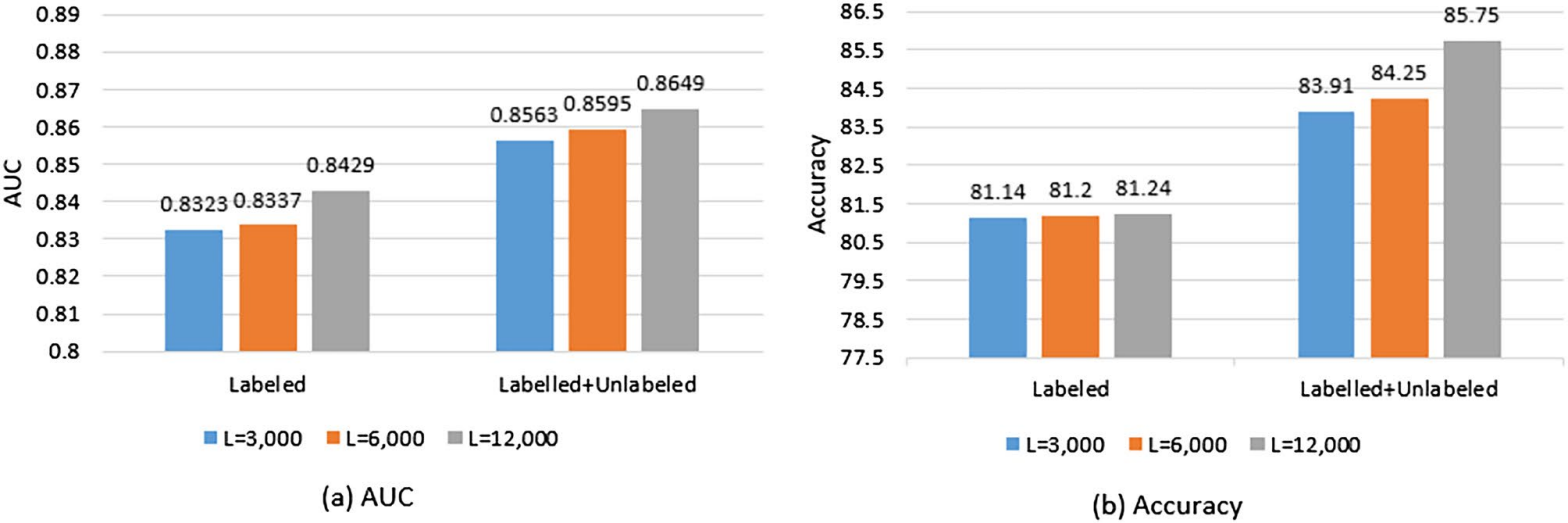
Neural network performance using labelled data only and mixed data(labelled and unlabelled) using different number of labelled data.

In the second experiment, we examine the impact of adding unlabelled data to $D_{train}$; we compare the performance of the neural network model found by NNI using different amount of originally labelled data and using labelled data combined with $D_{conf}$ in Fig. 7. The results show that increasing the number of labelled instances improves the network performance. Notably, the network performance using $D_{conf}$ combined with the labelled data outperforms that using the same amount of labelled data only. The performance of the network using $D_{train}$ only achieves an AUC of 0.8429, an accuracy of 81.24%, and F-score of 72.79% while the network achieves an AUC of 0.8649, accuracy = 85.75 and F-score = 77.29 using $D_{train}$ and $D_{conf}$.

In the third experiment, we compare the proposed semi-supervised approach to different supervised and semi-supervised baselines. For supervised baselines, we consider three classification networks for fully supervised learning; the network architecture found by NNI (used in the proposed approach), customized AlexNet^10^, a shallow VGG network^85^ modified to be fully convolutional and to also include batch-normalization^86^. The three fully-supervised baselines trained on $D_{train}$ only. The first semi-supervised baseline is DCGAN that involves a generative model trained along with a discriminator using both $D_{train}$ and $D_{conf}$. The discriminator is used to compute the loss of the classification, in addition to the adversarial loss^24^. The second semi-supervised baseline is self-training technique^87^ that leverages both labelled and unlabelled data for iterative self-training on pseudo-labeled predictions over task-specific unlabelled data. More specifically, we select three classic and well-known classifiers as self-labelled methods including decision tree, naive Bayes, and support vector machine. All of these selected base classifiers have been considered as one of the ten most influential data mining algorithms in^88^. We refer to self-training using a decision tree, naive Bayes and support vector machine as ST-DT, ST-NB, and ST-SVM, respectively. The configuration parameters of all the base classifiers used for the self-learning baseline are specified in Table 5.

**Table 5:** Parameter specification for all the base-learners used in the self-learning baseline used in the experimentation.

Algorithm	Parameter
ST-DT	Confidence level: c = 0.25, Mininum number of item-sets per leaf: i = 2, Prune after the tree building
ST-NB	No parameters specified
ST-SVM	C = 1.0, tolerance parameter = 0.001, Epsilon = 1.0 $\times$ 10–12, Kernel type = polynomial, Polynomial degree = 1, Fit logistic models = true

The semi-supervised baselines and our proposed approach are trained on $D_{train}$ and $D_{conf}$. In order to provide a fair comparison, the testing dataset used for testing the models performance is the same across all baselines networks and our proposed approach (50,963 patches).

**Table 6:** Performance comparison between different supervised and semi-supervised approaches.

	Supervised			Semi-supervised				
	VGG^85^	Customised AlexNet^10^	**NNI**	Semi-supervised DCGAN^24^	ST-DT^87^	ST-SMO^87^	ST-NB^87^	Our approach
F-measure	0.701 ${\pm }$ 0.003	0.6932 ${\pm }$ 0.001	**0.7297** ${\pm }$ 0.002	0.7421 ${\pm }$ 0.004	0.61 ${\pm }$ 0.010	0.621 ${\pm }$ 0.002	0.625 ${\pm }$ 0.005	**0.7729** ${\pm }$ **0.002**
Balanced accuracy	0.8235 ${\pm }$ 0.002	0.8121 ${\pm }$ 0.003	**0.8429** ${\pm }$ 0.001	0.8435 ${\pm }$ 0.005	0.721 ${\pm }$ 0.011	0.732 ${\pm }$ 0.001	0.741 ${\pm }$ 0.003	**0.8649** ${\pm }$ 0.001

Bold entry highlights the best-performing technique.

Underlined entry highlights the worst performing technique.

**Figure 8:**
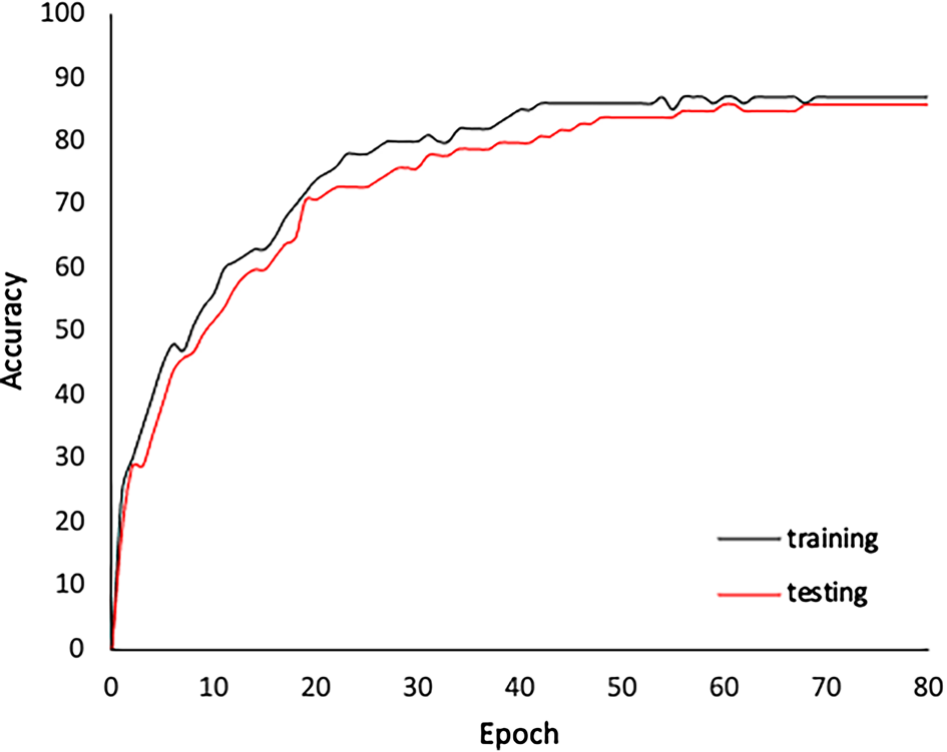
Training and testing accuracy of the network used in the proposed semi-supervised approach.

Table 6 shows the performance of different supervised and semi-supervised baselines. For each row metric and each category of baselines, we highlighted the highest performance in bold font and underlined the lowest performance. For the supervised baselines, the results show that the network used in the proposed framework outperforms AlexNet and VGG, achieving an F-measure of 0.7297 and balanced accuracy of 0.8429, while the supervised customised AlexNet baseline achieves the lowest performance (balanced accuracy of 0.8121 and F-measure of 0.6932). For the semi-supervised baselines, the results show that our proposed approach outperforms all semi-supervised baselines achieving a balanced accuracy of 0.8649 and F-measure of 0.7729, while the self-training baseline with decision tree base classifier achieved the lowest performance (balanced accuracy = 0.721 and F-measure = 0.61). Figure 8 shows the accuracy analysis for the training as well as testing. It is clear that the proposed approach achieves comparable performance on the training and testing datasets.

The Wilcoxon signed-rank test^83^ was conducted to determine if a statistically significant difference in terms of the performance exists between our proposed approach and all supervised and semi-supervised baselines. The results showed that the difference in performance between the proposed framework and all baselines is statistically significant with more than 95% level of confidence (p-value = 0.003).

#### Interpretability

We express the NHG criteria for nuclei pleomorphism as the average statistics of nuclei morphology and texture features. We compute some concepts from the nuclear segmentation dataset: average area, perimeter, Euler coefficient, axis length, and eccentricity. In addition, we calculate three of Haralick’s texture features^89^ including Angular Second Moment (ASM), contrast and correlation^89^. Nuclei pleomorphism features do not correlate with network prediction. Table 7, shows the correlation between the texture concepts measures and the prediction of the network found by NNI. Concept contrast has the most significant correlation coefficient of 0.44.

**Table 7:** Pearson correlation between concepts and network prediction.

	Correlation	ASM	Contrast
Correlation coefficient	${\textbf {-0.38}}$	0.32	**0.44**
*P*-value	$\le 0.001$	$\le 0.001$	$\le 0.001$

Significant values are in bold.

**Figure 9:**
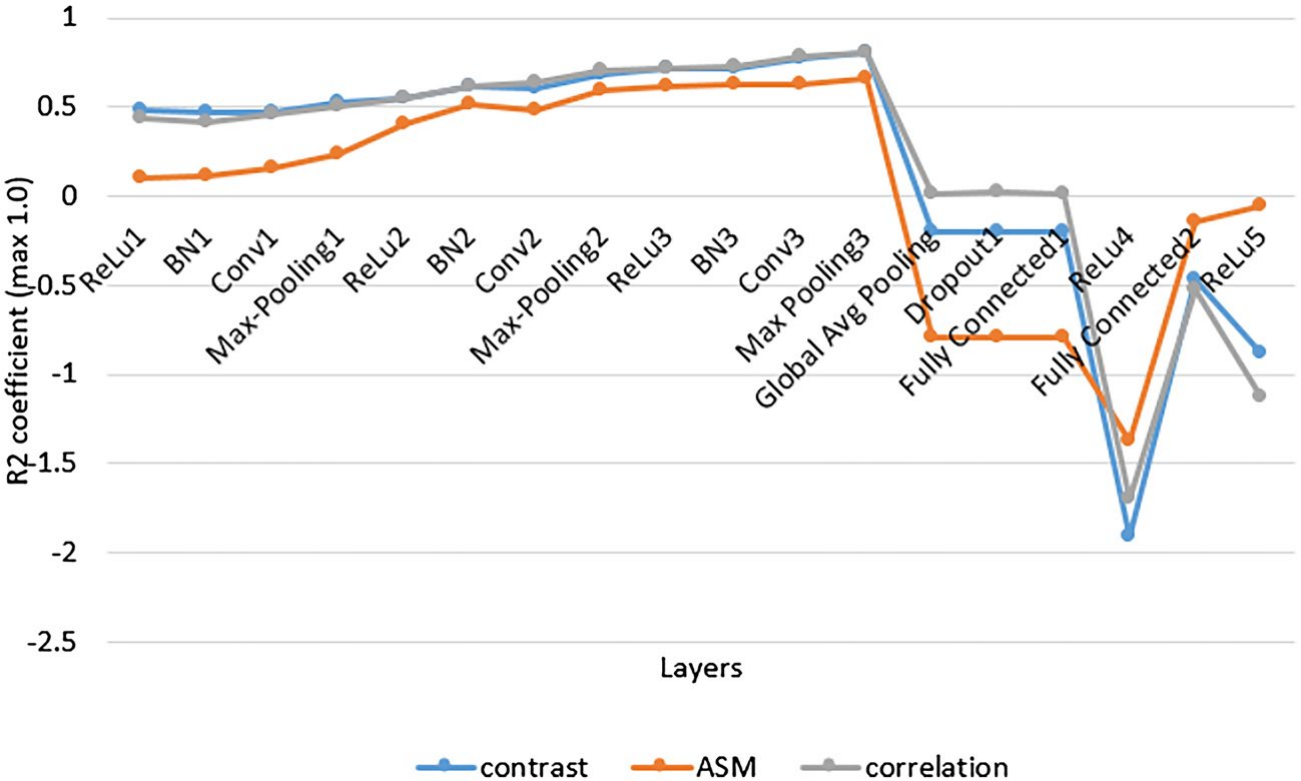
Determination coefficient of linear regression at all layers found by NNI.

To identify the network layer in which the concepts are learnt, we measure the performance of each linear regression model at each layer. The determination coefficient of the regression $R^2$ expresses the percentage of variation of the regression capture. For all patches in the testing dataset, we compute $R^2$ over multiple reruns to analyze the learning dynamics. More specifically, we compute $R^2$ using 10-fold validation averaged across 50 runs. The results show that all of the three concepts are learnt at the last Max-pooling layer, as shown in Fig. 9.

**Figure 10:**
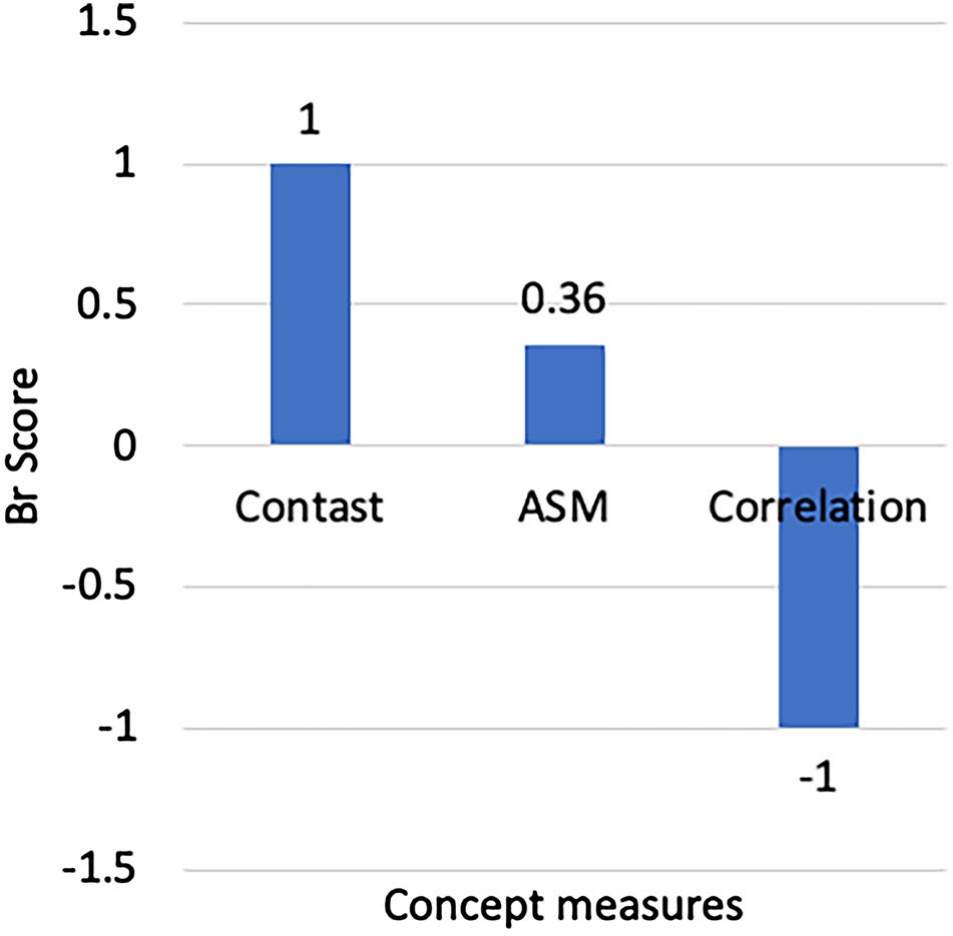
Br Score for texture concepts.

We compute the sensitivity for each test patch in the testing dataset. The global relevance is tested with *Br* score as shown in Fig. 10. Concepts contrast and correlation have *Br* scores of 1 and -1, respectively. These values are in line with the values of Pearson correlation in Table 7. The *Br* scores of the concepts show that contrast is relevant to classification, which aligns with the NHG grading system that identifies hyperchromatism as the leading cause of nuclear atypia. The *Br* score signs show that concept correlation negatively contributed to the prediction of IDC while concept contrast positively contributed to the prediction of IDC. In other words, if the correlation concept increases significantly, a patch may change from high risk of IDC to low risk of IDC. To consider that the resulting concepts are related to the class prediction in a significant way, we performed a two-tailed t-test to compare the distributions of the *Br* scores against the null hypothesis of learning a random direction for the *Br* (mean = 0) scores. The results show that there is a significant difference (with p-value = 0.001) in the scores for all the relevant concepts, namely correlation, ASM, and contrast.

## Discussion

In this study, we presented an interpretable semi-supervised deep convolution neural network model for IDC detection that uses a large amount of unlabelled data to improve the model performance. The availability of abundant labelled data for supervised learning is a challenging problem, especially in the medical domain. Most of the current techniques for abnormality detection requires manually annotated images for training. We developed a labelling technique to best utilize the labelled instances to label the unlabelled data and then append them to the small set of labelled instances to train a neural network model for IDC detection. Such labelling technique uses only a small amount of labelled data and the synthetic data generated by DCGAN. Using such a labelling technique, our neural network model achieves a balanced accuracy of AUC = 0.865 and an accuracy of 85.75% compared to a balanced accuracy of 0.843 and an accuracy of 81.28% when using only $D_{train}$. To achieve a high labelling performance, we tested different feature extraction techniques, including PCA, LDA and VGG-16. VGG-16 features achieve the best labelling performance (AUC = 0.815). Besides, we examine the labelling performance by testing the effect of increasing the size of the training dataset by generating synthetic patches using DCGAN. Results show that increasing the percentage of synthetic patches to 100% increases the labelling performance to an AUC of 0.847 and accuracy of 83.63% compared to an AUC of 0.815 and accuracy of 81.51% without using synthetic patches. It is concluded that the amount of unlabelled instances has a significant impact on the model performance, as shown in Fig. 7. Splitting the initially labelled dataset is an essential part of our labelling technique. Hence, if the size of the initially labelled dataset is extremely small (3k patches), then the ability to incorporate significant information from the unlabelled data would be very limited, as shown in Fig. 7. That is because extremely small initially labelled instances result in smaller splits that are more sensitive to noise.

The success of deep learning in perceptual tasks is mainly due to its automation of the most time-consuming feature engineering process; hierarchical feature extractors are learned in an end-to-end fashion from data rather than manually designed. This success has been accompanied, however, by an increasing demand for architecture engineering, where increasingly more complex neural architectures are designed manually. As the search space of the network architectures and hyperparameters is huge, we employed an AutoML framework to find the best network architecture and hyper-parameters on our dataset. We examine this architecture on the whole originally labelled training and validation datset and compared to the state-of-the-art performance by Cruz-Roa et al.^62^ and Janowczyk et al. ^10^. The results show that the network architecture found by NNI (balanced accuracy = 0.87 and F-score = 79%) outperforms the best baseline by Janowczyk et al. ^10^ (balanced accuracy = 0.85 and F-score = 76%). It is clear that the unlabelled data can not replace the labelled data and using unlabelled data is just a supplement. Labelled data contains precise and accurate information obtained from radiologists compared to automatically labelled data, proven to improve performance.

Complex machine learning models such as neural network models are hard to understand their behaviour and hence may pose a problem in their adoption in critical domains due to trust reasons. To explain the behaviour of the neural network model, we provide a global interpretability technique that explains the model based on meaningful concepts extracted from the nuclear segmentation dataset. The results show that nuclei contrast and correlation are the most important concepts to the classification of patches of breast tissue. The results align with the NHG grading system, which identifies hyperchromatism as a signal of nuclear atypia. The approach is beneficial and provides great insights when the user knows the set of concepts precisely and has enough examples for each of these concepts. The technique is quite flexible as extending the set of analyzed concepts can lead to identifying other relevant concepts. In addition, the technique is also adaptable to explain the predictions locally. One way to achieve that is by identifying the closest patches to the patch being explained and then calculating the BR scores on these batches.

## Conclusion

We presented a novel interpretable semi-supervised learning approach for IDC detection that uses a small amount of labelled data and a relatively large amount of unlabelled data to train a neural network model. The results of our experimental evaluation show that the performance of the neural network model used in the proposed framework is improved when combining the unlabelled data to the originally labelled data. The proposed framework significantly tackles the challenge of deep learning models requiring a large amount of data for their training, which is not always easy to obtain, especially in the medical domain. Our labelling model utilizes synthetic images generated by DCGAN to improve the labelling performance. Our approach allows users to utilize unlabelled data in a deep learning training dataset and increase the overall performance. To build trust in the developed framework, we explain the neural network model globally using texture concepts extracted from the nuclear segmentation dataset. The main limitation of this interpretability technique is that the space for meaningful concepts to be queried is unlimited, and in some cases, it is hard to provide enough examples for each of these concepts. Another primary limitation is that querying a particular set of concepts may create a biased explanation process toward such provided concepts while failing to query the right set of concepts. One possible future direction is to extend our work and consider different interpretability techniques that can extract meaningful concepts automatically from that data. Another future direction is to expand the applications of our proposed approach to different histopathology problems and develop tools to be put in practical use to assist the pathologists with faster and more efficient diagnoses.

## Data Availability

For IDC detection, the batch-based dataset used in the current study is available at http://www.andrewjanowczyk.com/use-case-6-invasive-ductal-carcinoma-idc-segmentation/. The concepts used by the global interpretability technique are extracted from the nuclear segmentation dataset available at https://nucleisegmentationbenchmark.weebly.com/dataset.html. The implementation of the method, acknowledgement files for the IDC detection used is provided at https://github.com/DataSystemsGroupUT/DC-classification.
